# A label-free G-quadruplex-based mercury detection assay employing the exonuclease III-mediated cleavage of T–Hg^2+^–T mismatched DNA

**DOI:** 10.1088/1468-6996/16/6/065004

**Published:** 2015-11-17

**Authors:** Wanhe Wang, Tian-Shu Kang, Philip Wai Hong Chan, Jin-Jian Lu, Xiu-Ping Chen, Chung-Hang Leung, Dik-Lung Ma

**Affiliations:** 1 Department of Chemistry, Hong Kong Baptist University, Kowloon Tong, Hong Kong, China; 2 State Key Laboratory of Quality Research in Chinese Medicine, Institute of Chinese Medical Sciences, University of Macau, Macao, China; 3 School of Chemistry, Monash University, Clayton, Victoria 3800, Australia; 4 Department of Chemistry, University of Warwick, Coventry CV4 7AL, UK; 5 Partner State Key Laboratory of Environmental and Biological Analysis, Hong Kong Baptist University, Hong Kong, China

**Keywords:** iridium(III), exonuclease III, G-quadruplex, mercury(II) ion

## Abstract

We report herein the use of an exonuclease III and G-quadruplex probe to construct a G-quadruplex-based luminescence detection platform for Hg^2+^. Unlike common DNA-based Hg^2+^ detection methods, when using the dsDNA probe to monitor the hairpin formation, the intercalation of the dsDNA probe may be influenced by the distortion of dsDNA. This ‘mix-and-detect’ methodology utilized the G-quadruplex probe as the signal transducer and is simple, rapid, convenient to use and can detect down to 20 nM of Hg^2+^.

## 1. Introduction

Mercury ions (Hg^2+^) are a hazardous pollutant produced by refineries, factories, power station or runoff from landfills. Long-term absorption of mercury may result in kidney damage, memory impairment and other severe health problems [1, 2]. Living organisms will absorb and then accumulate mercury in fatty tissue via eating and drinking. Therefore, exposure of the human body to mercury should be minimized due to potential metal accumulation. According to the United States Environmental Protection Agency, the maximum safe concentration of mercury in drinking water is 2 ppb, the quantitative detection of which would require a very sensitive instrument [3–5]. Unfortunately, existing analytical methods for the sensitive detection of Hg^2+^ are not satisfactory for in-field use due to expensive instrumentation and labor-intensive sample preparation protocols. These instrumental methods include atomic absorption spectroscopy (AAS), inductively-coupled plasma mass spectrometry (ICP-MS) [6] and ion-selective electrodes [7, 8].

In 2004, Ono and co-workers discovered that Hg^2+^ is able to coordinate with thymine nucleobases [9–12]. This discovery has sparked a wave of novel Hg^2+^ detection methods, such as oligonucleotide-based luminescence [13–28], colorimetric [29–40], electrochemical [41, 42] and surface-enhanced Raman scattering methods [43–46]. Later on, they reported the crystal structure of a DNA duplex containing the T−Hg^II^−T mismatch, which showed that the T−Hg^II^−T bond largely distorts DNA structure and also stabilizes B-form DNA [12]. Moreover, C−Ag^I^−A [47] and C−Ag^I^−T mismatches have also been reported [11].

The G-quadruplex is a DNA secondary structure which is formed from a guanine-rich DNA sequence with four or more G-tracts. It is stabilized by monovalent cations such as K^+^, Na^+^ or NH_4_^+^ to form square-planar arrangements of guanine residues [48, 49]. Because of its rich structural polymorphism, the G-quadruplex has been widely used to construct analytical detection platforms [43, 44, 50–64] or various types of logic gates [65–67].

Exonuclease III (ExoIII) is a nuclease that catalyzes the stepwise removal of mononucleotides from the 3′-terminus of duplex DNA. Exo III has been used as a component of oligonucleotide-based detection platforms for different targets or as a tool for signal amplification [68, 69]. Interestingly, ExoIII has been reported to cleave DNA duplexes containing T−Hg^2+^−T mismatches and release Hg^2+^ into solution [42].

In our previous study, we constructed AND, OR and INHIBIT logic gates utilizing Hg^II^ and Ag^I^ ions as signal inputs. The ‘Klenow fragment’ polymerase was utilized as one of the signal transducing elements that catalyzes the extension of designed DNA in the 5′ to 3′ direction to form a duplex product and displace the split G-quadruplex sequence in the presence of Ag^+^ and Hg^2+^ [70]. Moreover, previous investigations demonstrated that the photophysical properties of iridium(III) complexes could be fine-tuned by changing the CˆN or NˆN donor ligand. We report herein a novel detection platform for Hg^2+^ using a novel iridium(III) complex. Unlike common detection methods using dsDNA probes to monitor the hairpin formation, which could be influenced by the distortion of dsDNA by mismatches, our approach utilized a G-quadruplex probe and is based on the newly discovered phenomenon that ExoIII cleaves DNA duplexes containing T−Hg^2+^−T mismatches. The detection mechanism of this platform is outlined in scheme 7. The DNA probe contains a G-quadruplex-forming sequence (green line), and its complementary sequence (red line), as well as 3′ and 5′ polyT (poly thymine) overhangs. The overall DNA structure assumes a hairpin formation with unhybridized 3′ and 5′-termini. Since ExoIII is unable to recognize single-stranded DNA (ssDNA) as substrate, it will not cleave the unhybridized 3′ overhang and the hairpin DNA will not be digested in the absence of Hg^2+^. Upon exposure to Hg^2+^ however, the 3′ and 5′ polyT overhangs form T−Hg^2+^−T bridges, creating a duplex DNA structure that is vulnerable to ExoIII digestion from the 3′-terminus (red line). ExoIII cleavage releases the G-quadruplex-forming sequence. The G-quadruplex structure binds with the luminescent G-quadruplex-selective iridium(III) complex **1**, with a switch-on emission response. Furthermore, the Hg^2+^ may be recycled as they are re-released into solution following the ExoIII cleavage step.

**Scheme 1.**
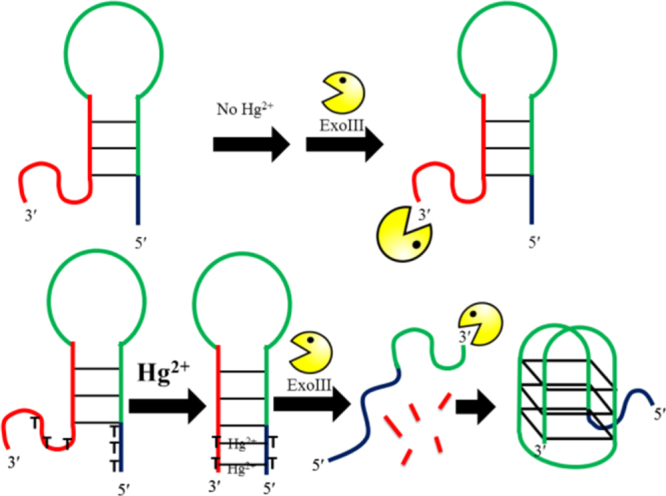
Schematic representation of the ExoIII-assisted label-free G-quadruplex-based assay for Hg^2+^.

## 2. Experimental details

### 2.1. Materials

Reagents, unless specified, were purchased from Sigma Aldrich (St. Louis, MO, USA) and used as received. Iridium chloride hydrate (IrCl_3_ · xH_2_O) was purchased from Precious Metals Online (Australia). All oligonucleotides were synthesized by Techdragon Inc. (Hong Kong, China).

### 2.2. Synthesis

Complex **1** was prepared according to (modified) literature methods and was characterized by proton nuclear magnetic resonance (^1^H-NMR), ^13^C-NMR and high resolution mass spectrometry (HRMS). Complex **1**. ^1^H NMR (400 MHz, acetone-*d*_6_) *δ* 8.80 (*d*, *J* = 8.8 Hz, 2H), 8.53 (*d*, *J* = 8.8 Hz, 2H), 8.14-8.09 (*m*, 4H), 7.89 (*d*, *J* = 8.8 Hz, 2H), 7.83-7.81 (*m*, 2H), 7.67-7.63 (*m*, 2H), 7.42-7.38 (*m*, 2H), 7.31-7.27 (*m*, 2H), 7.21-7.17 (*m*, 2H), 7.15-7.06 (*m*, 6H), 6.58 (*d*, *J* = 7.6 Hz, 2H); ^13^C NMR (100 MHz, DMSO-*d*_6_) *δ* 181.0, 161.3, 148.9, 148.8, 147.8, 142.1, 140.0, 132.5, 132.1, 131.7, 131.3, 129.9, 129.7, 129.6, 128.5, 127.5, 127.3, 126.9, 125.0, 123.9, 122.1, 118.1; HRMS: calculated for C_44_H_28_IrN_4_S_2_ [M–PF_6_]^+^ 869.1385; found 869.1332; analyzed (C_44_H_28_IrN_4_S_2_PF_6_). C, H, N: calculated 52.12, 2.78, 5.53; found 52.34, 2.77, 5.58.

## 3. Results and discussion

**Figure 1.**
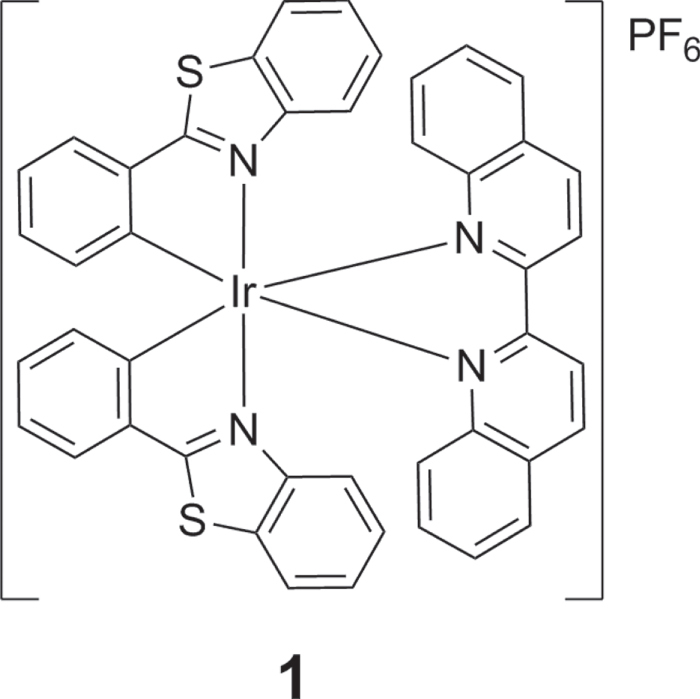
Chemical structure of cyclometalated iridium(III) complex **1**.

**Figure 2.**
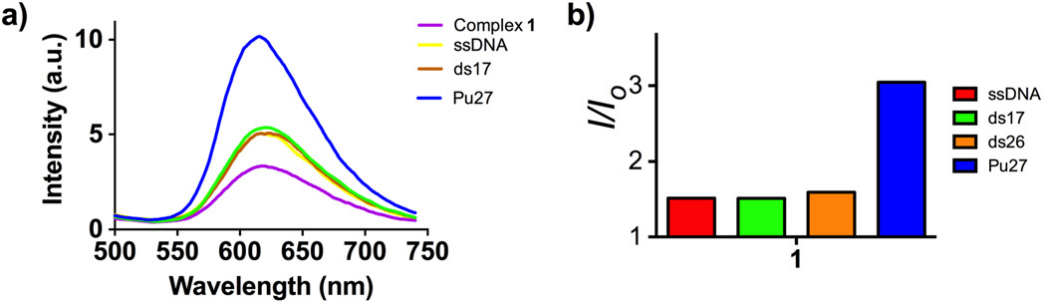
(a) Emission spectrum and (b) luminescence enhancement of complex **1** (1 *μ*M) in the presence of 5 *μ*M of ssDNA, ds17, ds26 or Pu27 G-quadruplex.

**Figure 3.**
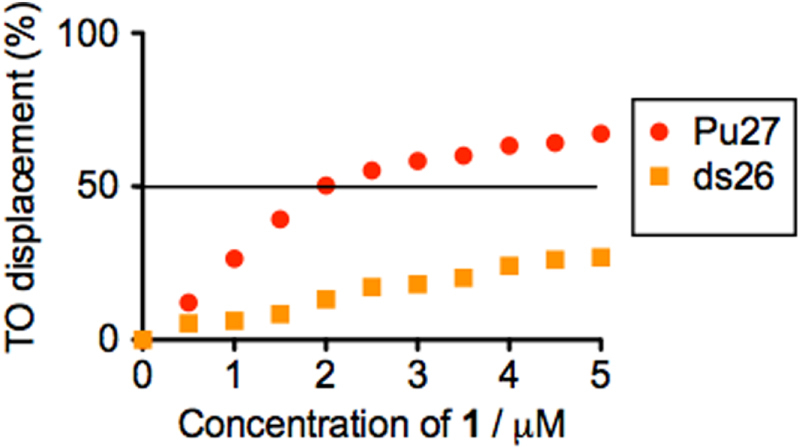
Percentage of TO displaced from DNA duplex ds26 or Pu27 G-quadruplex upon addition of complex **1**.

Complex **1** is a cyclometalated iridium(III) complex: [Ir(pbt)_2_(biq)]PF_6_ (**1**, where pbt = 2-phenylbenzo[*d*]thiazole, biq = 2,2′-biquinoline, figure 1). The synthesis, characterization and photophysical properties of **1** are given in the supplementary information (table S1, figure S1). No significant change in the UV–visible absorption spectrum of **1** was observed in aqueous buffered solution over 48 h, indicating that the complex is stable in aqueous solution and may be suitable for long term use. By itself, complex **1** was weakly emissive in aqueous buffered solution (20 mM Tris, pH 7.0). Encouragingly, we found that a significant increase in luminescence response of **1** can be triggered by the presence of G-quadruplex DNA, as shown by emission titration experiments. We then explored the effect of the addition of various types of DNA (figure 2). ssDNA or double-stranded DNA (ds17, ds26) had only a minor effect on the luminescence of **1**. However, in the presence of the Pu27 G-quadruplex, the luminescence of **1** was significantly increased (about 3-fold). We anticipate that the G-quadruplex shields the metal center of **1** from solvent quenching by non-radiative decay of the excited state, thus recovering ^3^MLCT phosphorescence. To validate this hypothesis, the G-quadruplex fluorescent intercalator displacement (G4-FID) assay was utilized to investigate the binding affinity of **1** for G-quadruplex and dsDNA. The result showed that only 2 *μ*M of **1** could displace 50% of thiazole orange (TO) from G-quadruplex-TO assemble. On the other hand, only about 30% of TO could be displaced from dsDNA-TO assemble in the presence of 5 *μ*M of complex **1** (figure 3). It outlines the G-quadruplex selective property of complex **1**.

**Figure 4.**
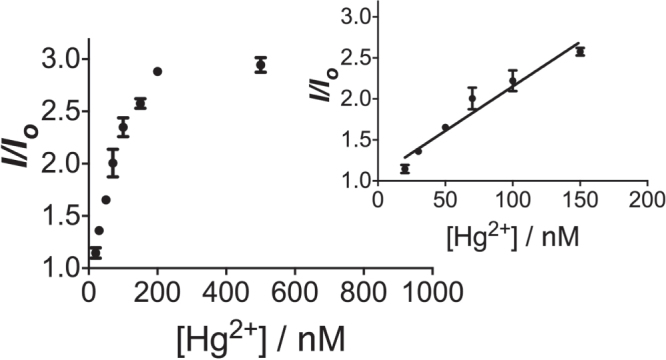
Emission enhancement of the system ([**1**] = 1 *μ*M, [DNA] = 1.5 *μ*M, [K^+^] = 100 mM) with increasing concentration of Hg^2+^.

As complex **1** displays the property of a G-quadruplex-selective probe, we sought to apply it in our Hg^2+^ sensing platform. Encouragingly, we observed that the presence of Hg^2+^ enhanced the luminescence intensity of the complex **1**–hairpin DNA system (figure 4). To validate that the luminescence enhancement is due to the switching of the DNA structure, we also sought to exclude the possibility of direct interaction between complex **1** and Hg^2+^ as a contributor to the response. We found that no luminescence increase was observed for the system lacking hairpin DNA (figure S2). We propose that the observed luminescence enhancement of the system is due to the formation of the T−Hg^2+^−T mismatched duplex in the 3′ and 5′ overhangs, which allows ExoIII digestion of the hairpin DNA, resulting in the release of the G-quadruplex-forming sequence and the formation of a G-quadruplex structure that is recognized by the G-quadruplex-selective complex **1** with a switch-on emission response.

**Figure 5.**
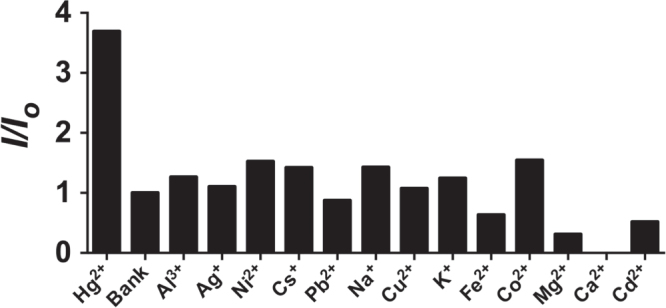
Relative luminescence intensity of the system ([**1**] = 1 *μ*M, [DNA] = 2 *μ*M) in the presence of 10 nM Hg^2+^ or 5-fold excess of other metal ions.

The linear detection range of the system was found to be from 20 to 200 nM of Hg^2+^ (figure 4). Maximal luminescence was reached at 200 nM. The detection limit of the present assay was determined to be 20 nM of Hg^2+^ by the 3*σ* method. The selectivity of this detection platform for Hg^2+^ over other metal ions was also evaluated. The results showed that the luminescence response of the system for Hg^2+^ was significantly stronger than that for 5-fold excess concentrations of the other metal ions (figure 5).[Fig F0006]

**Figure 6. F0006:**
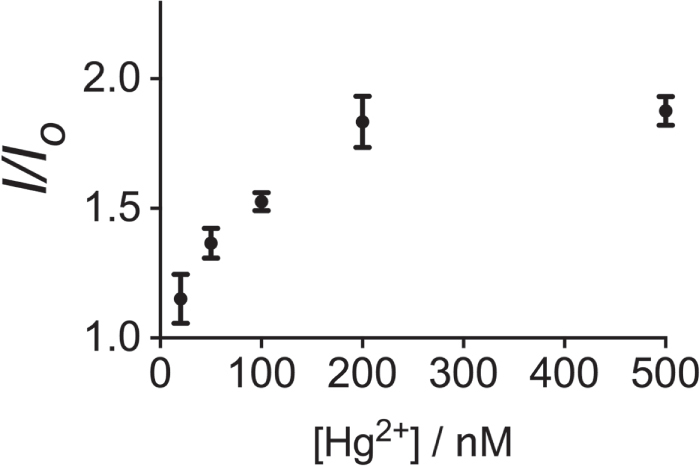
Luminescence response of the system to increasing concentrations (20, 50, 100, 200 and 500 nM) of Hg^2+^ in a 50-fold diluted river water sample.

In order to investigate the effectiveness of this Hg^2+^ assay in a practical application, we applied it to the detection of Hg^2+^ in spiked natural water samples. No Hg^2+^ was detected in the natural water samples by ICP-MS. Therefore, the natural water samples (Nam Sang Wai River in Hong Kong) were diluted 50-fold with Tris buffer and spiked with various concentrations (20, 50, 100, 200 and 500 nM) of Hg^2+^. The samples showed a gradual increase in luminescence intensity with Hg^2+^ (figure 6). This result demonstrates that our detection platform could potentially be further developed as a sensitive probe for natural water sample analysis of Hg^2+^[Fig F0006].

## 4. Conclusions

In conclusion, a novel luminescent iridium(III) complex which shows the G-quadruplex probe property, was investigated and utilized for the construction of an exonuclease-assisted, label-free G-quadruplex-based luminescence detection platform for Hg^2+^. Compared with the modified DNA, the cost label-free approach using unmodified DNA is relatively low. Unlike common detection methods, when using the dsDNA probe to monitor the hairpin formation, the intercalation of the dsDNA probe may be influenced by the distortion of dsDNA. This ‘mix-and-detect’ methodology utilized the G-quadruplex probe as the signal transducer and it is simple, rapid, convenient to use and can detect down to 20 nM of Hg^2+^, which is comparable to recently reported label-free DNA-based Hg^2+^ ion detection methods. For comparison, we have also summarized those reported methods in table S2. Furthermore, the potential application in a real water sample was also demonstrated, which shows the robustness of the system.
